# Patient Delay in Accessing Breast Cancer Care in a Sub Saharan African Country: Uganda

**DOI:** 10.9734/BJMMR/2014/7293#sthash.PglzE4b6.dpuf

**Published:** 2014-05-01

**Authors:** Moses Galukande, Florence Mirembe, Henry Wabinga

**Affiliations:** 1 Moses Galukande, Department of Surgery, College of Health Sciences, Makerere University, Uganda.; 2 Florence Mirembe, Department of Obstetrics and Gynaecology, College of Health Sciences, Makerere University, Uganda.; 3 Henry Wabinga, Department of Histopathology, College of Health Sciences, Makerere University, Uganda.

**Keywords:** Patient delay, breast cancer, late presentation, Uganda

## Abstract

**Aims:**

To assess patient delay differences between early and late stage breast cancer among women in Uganda.

**Study Design:**

A retrospective analytical study.

**Place and Duration of the Study:**

A study conducted at a tertiary teaching hospital. Selected patients’ data available for the period between 2008 and 2011 were included in this study.

**Methodology:**

We included 201 women with histologically confirmed breast cancer. The variables analysed included age, residence, histological subtype, stage at presentation and time delays. Ethical approval was obtained.

**Results:**

The mean age for the early and late presenters was 49 and 46 years respectively (p=0.065). Rural women were more likely to present late. Triple negative breast cancer (TNBC) and HER2+ were the majority cancer subtypes for the late presenters. On average women waited for 29 months before they presented for specialized cancer treatment (median 12 months; range 1-120 months). The duration of symptoms didn’t differ between the two groups (p=0.295) and 75% of early stage presenters, reported at least 6 months after noticing symptoms. Only 9% of the TNBC patients presented under 3 months in comparison to 14 % for HER2+, 33% for Luminal B and 36% for luminal A. Overall 23% (39/168) presented with early stage disease.

**Conclusion:**

Delay in seeking appropriate breast cancer care in Uganda was excessive, a sign of a neglected disease. Tumor biology factors seem to play a role in late stage presentation. Research in factors that lead to prolonged delay in accessing care in a resource poor context are needed urgently.

## 1. INTRODUCTION

Breast cancer is the most common cancer among women and the main cause of cancer-related deaths worldwide; causing approximately 2-million new cases and 500,000 deaths in 2008 [1]. It is the second main cause of non HIV cancer-related deaths among women in Uganda [2,3].

In developing countries, breast cancer survival rates are much lower than in developed countries, mainly because cancer is diagnosed in later stages. In the United States, 60% of breast cancer cases are diagnosed in stages 0 and I, with survival rates of 98% [4]. Whereas in Uganda less than 20% of patients are diagnosed in these early stages and more than 80% in the most advanced stages (III and IV) [2,5]. The main reasons for presentation of breast cancer patients in advanced stages could be related to the lack of access to breast cancer screening [5,6] delayed help-seeking for breast cancer symptoms and barriers to accessing health care services [7]. In addition it may be due to tumor biology factors [8,9] and lack of awareness [10].

Breast cancer delay is defined in the literature as a span of more than three months between the discovery of symptoms by the patient and the beginning of definitive cancer treatment [7]. Traditionally, it has been classified in two types: patient and provider delay. Cut-off points to define these intervals vary across studies, but the majority of studies have considered patient delay to be more than three months between the discovery of symptoms and the first medical consultation [11-13]. In turn, provider delay takes place between the first medical consultation and the beginning of definitive treatment, and the most accepted threshold is one month, although this cut- off point varies across studies [12,14].

There is a dearth of data in sub-Saharan Africa on the subject of delay for breast cancer patients; in a recent literature review only 5 studies were available from developing countries [7].

The purpose of this study therefore was to assess the factors associated with delay in a group of women with breast cancer in Uganda.

## 2. MATERIALS AND METHODS

### 2.1 Study Design

This was a retrospective analytical study.

### 2.2 Setting

Mulago Hospital, the teaching hospital for Makerere College of Health Sciences and the Uganda Cancer Institute in Uganda. Mulago Hospital is Uganda’s national referral hospital located in Kampala the capital city. It is a 1500 bed hospital and runs a specialized breast clinic where approximately 5 new breast cancer patients are seen every week. The breast clinic runs once a week.

### 2.3 Sampling

Consecutive for patients’ data that were available for the period between 2008 -2011, patient files with insufficient clinical data were excluded from the analysis.

### 2.4 Study Procedures

Clinical staging was done based on physical findings of tumor size, nodal status supplemented by breast ultrasonography, a chest x-ray, an abdominal scan and bone scans for those symptomatic for bone metastases. Laboratory procedures have been previously described [5].

### 2.5 Study Variables

Age, occupation, stage and duration of symptoms; the duration of symptoms from the time the patient noticed symptoms in the breast to the first time of presenting to the national referral hospital; the only public cancer treatment centre offering largely free care. The other study variables were phenotypes and area of residence (rural or urban).

### 2.6 Analysis

SPSS 17 software was used, descriptive for frequencies, chi square tests for comparison of variables and significance was when p=/< 0.05.

## 3. RESULTS AND DISCUSSION

### 3.1 Results

A total of 201 patient data were included in the analysis. Table 1 shows the demographic characteristics of the participants in the study.

In Table 2, a comparison is made between the early stage and late stage presenters.

The mean age for early stage presenters was 49 years whereas for the late stage presenters was 46, with borderline statistical significance (p= 0.065).

Most of the women came from the rural areas and the bulk of the late stage presentations were rural.

Triple negative breast cancer (TNBC) contributed the majority of late disease presenters; there was a 10-fold difference between luminal and TNBC tumors.

The duration of symptoms didn’t differ between the two groups of early and late presentations (p=0.295), and 75% of early stage presenters, reported at least 6 months after noticing symptoms. Over all, only 3 patients present within 3 months and with early stage disease. The other 6 patients that presented within 3 months had late stage disease.

Only 9% of the TNBC patients presented with early stage disease in comparison to 14 % for HER2+, 33% for Luminal B and 36% for luminal A, overall 23% (39/168) presented with early disease.

Those that presented within 3 months of noticing symptoms by subtypes were 4% (6/157) for

TNBC, 41% (6/157 for luminal B and 2% (2/157) for HER2+.

Those that presented within 6 months of noticing symptoms; by subtypes were 28% for

TNBC, 21% for HER2+, 19% for Luminal A and 9% for Luminal B (see above Table 3).

### 3.2 Discussion

In this study we set out to investigate the differences between breast cancer patients that presented with early stage (I & II) cancer and those that presented with late (stages III & IV)

We found that the mean delay was 29 months, a small proportion of women with early disease presented within 3 months of noticing symptoms. However, more than 75% of the early stage presenters reported more than 6 months after noticing symptoms.

Early stage presenters were slightly older than the late stage presenters by three years, with borderline statistically significance (p=0.065). In a paper by Burgess, 2006, it was suggested that the older a woman was, the more likely that they present with late stage disease. It has also been suggested that breast cancer tumors grow faster in younger women and therefore likely to contribute to late stage presentation [15]. This perhaps in part explains the diversion from the previous notion that older women are likely to present with late stage disease.

More late presenters had mostly TNBC and HER2 tumors compared to those with luminal; this may be due to differences in factors that drive tumor growth. It could be that tumor doubling time (growth rate) for TNBC is shorter [8,16]; in part explaining why there wasn’t such a time difference for the duration of symptoms in both groups. It also appears from these data that TNBC tumors were less likely to be self detected. Could it be that they were more subtle in presentation or more elusive to self detection? Could this be a distinct clinical characteristic of TNBC? Comparatively more late presenters were rural women, rural dwellings are a contributor to poor access to care due to geographical and socioeconomic barriers [17,18], though it was not statistically significant in this study.

The majority of breast cancer deaths occur in developing countries [19]. Mortality reductions achieved in the last decades in developed countries have not been achieved in developing countries mainly because of a lack of access to early medical attention [20,21].

Most cancer in low- and middle-income countries (LMC) is detected at later stages [2,22]. It is commonly assumed that this late diagnosis is due to the populations’ lack of information and deficient or absent screening programs.

In this study some patients had waited for over 100 months; it is possible that they had very slow growing tumors or had benign conditions upon which a malignancy was later superimposed, but the real reasons for this wait will be crucial to design appropriate interventions.

Most studies have found that the longer the delay, the more likely a woman is diagnosed in advanced stages and therefore lower survival rates [11,13,23-25]. The most likely explanation for the association between delay and survival is that delay allows disease progression [13]. Various other studies yielded contradictory findings as described in the Unger –Saldana article in 2009 [7]. The author explained that differences in conclusions between studies may have been due to: differing sample characteristics (including patients in all clinical stages or only patients with operable cancer), differences in the delay interval studied (patient, diagnostic, treatment, provider, total delay or different combinations) and differences in time periods used to define delay.

Breast cancer tumor doubling time is about 130 days (3 months) [9] assuming linear growth and assuming a breast tumor will be palpable at 25mm; a 6 months wait will inevitably allow a 25mm tumor to grow to 45mm tumor size. The mean delay period in this study was 29 months (median 12 months), we would theoretically expect nearly everyone to be at stage III and IV.

While for many patients, delays between three and six months would probably not have an impact on 5-year survival, it has been well documented that as delay time increases, so does the probability of clinical progression, which has been shown to negatively affect survival [26].

In this study the majority of women discovered their own lumps through non-routine incidental circumstances. Self detection is mostly possible when the tumor size is about 2.5 cm [26]. It is also easier to find if it is relatively close to the skin and with the tumor to breast size ratio is in favour. Tumors at 25 mm are technically stage II or beyond. The challenge remains as to what other possibilities are available to getting women present earlier to the appropriate points of care in the context of non-existent breast cancer screening programs. The BSE and CBE practices are currently not supported by evidence [27,28]. Innovative low cost technologies may be the way forward [29], exploring the use of the breast light at community level maybe one of such ideas [30,31]. Creating awareness at village community level recently piloted in Sudan deserves attention [32].

Whereas previously published work relates delay mostly to socio economic factors and the stage of the disease, we highlight a link to tumor biology. It appears that tumor biology is a major contributor to late stage presentation. When all the patients take the same time to come to hospital, those with TNBC will have more advanced tumors. A study investigating delay had never been documented before for Uganda. This study also highlights the severity of access limitation to specialized breast care irrespective of the underlying reasons

The absence of early detection and access to care in developing countries such as Uganda should be looked at as an ethical issue.

### 3.3 Limitations

We were unable to find out the patients perception of their symptoms the first time they noticed them, as this would have impacted on the action they took or did not take. We hypothesize that many may not have considered their findings life threatening [26].

What we did not take into account was the fact that some patients were likely to have sought alternative care (such as traditional healers) before coming to hospital which could be a factor that contributes to delay and needs to be quantified.

The current staging systems are not foolproof; some patients may appear with small tumors but carry undetected metastases [33,34].

Even though this is a single country study, many countries in East, Central and Southern Africa share similar socio-economic and cultural contexts.

## 4. CONCLUSION

Delay was excessive for both late and early stage presenters. Identification of underlying modifiable factors and the appropriate interventions to mitigate prolonged delay are needed urgently.

## Figures and Tables

**Table 1:** Client characteristics for early and late stage breast cancer presenters, Uganda delay study Variable

Variable	N	%
mean age	46.5Y, SD 13 (95%CI44-48)	
median age	45 Y	
range	22-87	
age category*		
<30	20	10
31-40	62	31
41-50	52	26
51-60	39	20
>60	25	13
missing	4	13
duration of symptoms		
mean (months)	29Y, SD 34 (95% CI 20-30)	
median	12	
range	1-120	
occupation		
peasant	65	38
house wife	36	21
business	32	18
formal employment	37	21
unemployed	3	2
missing	29	
setting		
rural	139	72
urban	55	28
missing	8	
missing	65	
means of problem detection (discovery)		
BSE	108	79
CBE	5	4
others	24	17
missing	61	
prompts incidental	128	90
routine exam	8	6
other	7	4
tumor stage (clinical)		
I	15	8
II	32	17
III	126	65
IV	20	10
missing	9	
phenotypes		
Luminal A	75	43
Luminal B	12	7
HER2+	35	21
TNBC	50	29
missing	30	
Family history of breast cancer		
yes	45	26
no	122	72
don’t know	4	2
missing	31	
duration of symptoms		
< 3months	16	9
3-6 months	21	11
6-12	46	25
12-24	50	28
>24	49	27
missing	20	

**Table 2:** Comparing participants’ characteristics for early and late stage presenters, Uganda delay study

Variable	Early stage	Late stage	p value
age	n=45 (23)	n=49 (77)	
mean in years	49y(sd14)	46y(sd13)	0.065
median	49y	42y	
range	25-80y	25-87y	
age categories			
< 30	4	14	0.786
31-40	12	46	
41-50	10	40	
51-60	9	30	
>60	8	17	
duration of symptoms			
mean	19 (sd 15)	18 (sd 32)	0.295
median	12	12	
duration of symptoms categories			
<3 months	3	13	<0.001
3-6 months	5	16	
6-12 months	10	36	
12-24 months	11	36	
> 24 months	10	37	
setting			
rural	33	101	0.585
urban	11	41	
Subtypes			
Luminal A	26	45	<0.001
Luminal B	3	8	
HER2+	5	31	
TNBC	4	44	

**Table 3:** The distribution of duration of symptoms (in months) and stage by phenotypes, Uganda delay study, 2013

Duration of symptoms	Over all	Early stage n=34	Late stage n=139	Luminal A n=68	Luminal B n=12	HER2+ n=33	TNBC n=45	Missing n=25	P value
Mean duration	29	21	23	26	17	19	21	23	-
Median duration	12	12	12	21	24	12	12	24	
SD	26	21	28	30	10	18	33	16	
Percentiles									
25	7	6	7	8	6	7	6	9	-
50	12	12	12	21	24	12	12	24	
75	24	24	30	34	24	27	24	36	
Missing	-	6	10	7	-	2	5	-	
Duration categories									0.370
<3 months				6	0	2	6		
3-6 months				7	1	5	7		
6-12 months				12	3	12	12		
12-24 months				24	6	6	12		
>24 months				19	1	9	9		
Total				68	11	34	46		
Duration by stage at diagnosis									
I				8	1	1	2	<0.001	
II				18	3	4	2		
III				41	6	26	37		
IV				6	2	5	6		
Total				73	12	36	47		

## ABBREVIATIONS

BSE: Breast Self Examination
CBE: Clinical Breast Examination
TNBC: Triple Negative Breast Cancer
